# Long-Term Coronary Artery Disease Risk Prediction with Machine Learning Models

**DOI:** 10.3390/s23031193

**Published:** 2023-01-20

**Authors:** Maria Trigka, Elias Dritsas

**Affiliations:** Department of Computer Engineering and Informatics, University of Patras, 26504 Patras, Greece

**Keywords:** healthcare, long-term risk prediction, machine learning, coronary artery disease, feature analysis

## Abstract

The heart is the most vital organ of the human body; thus, its improper functioning has a significant impact on human life. Coronary artery disease (CAD) is a disease of the coronary arteries through which the heart is nourished and oxygenated. It is due to the formation of atherosclerotic plaques on the wall of the epicardial coronary arteries, resulting in the narrowing of their lumen and the obstruction of blood flow through them. Coronary artery disease can be delayed or even prevented with lifestyle changes and medical intervention. Long-term risk prediction of coronary artery disease will be the area of interest in this work. In this specific research paper, we experimented with various machine learning (ML) models after the use or non-use of the synthetic minority oversampling technique (SMOTE), evaluating and comparing them in terms of accuracy, precision, recall and an area under the curve (AUC). The results showed that the stacking ensemble model after the SMOTE with 10-fold cross-validation prevailed over the other models, achieving an accuracy of 90.9 %, a precision of 96.7%, a recall of 87.6% and an AUC equal to 96.1%.

## 1. Introduction

The heart is a tireless muscular pump, the size of a large fist and weighing 300–400 g. It circulates tons of blood during human life. Cardiovascular disease remains the leading cause of death despite significant advances in medical science. It needs special attention and awareness to minimize the factors that cause it, as nowadays, the habits and lifestyle of modern people directly impact it [1,2].

The coronary arteries are the arteries that transport blood to the heart muscle and supply it with the necessary ingredients for its function. The term “coronary artery disease” is used to describe the narrowing of these arteries, which is caused by the accumulation of atherosclerotic material in their lumen. Due to the stenosis, the heart muscle is not adequately supplied with blood–especially in situations where it has increased needs–and this causes myocardial ischemia [3]. In the vast majority of cases, CAD is caused by the progressive accumulation of atherosclerotic material, which narrows the lumen of the arteries and causes myocardial ischemia. Atherosclerotic material is a soft, fatty material that forms on the inner surface of the arteries by interacting with blood elements (cells and coagulation factors) and fats carried by the blood. Atherosclerotic plaque “hardens” over the years due to calcium deposition [4].

Angina pain is a common manifestation of insufficient perfusion of the myocardium, and manifests in discomfort in the centre of the chest which may be tight, or feel like burning or pressure. Angina may be felt in both hands, in the area of the neck, lower jaw, in the mid-shoulder area and in the epigastrium. Sometimes, when the pain is intense, sweat, nausea or vomiting occur. Manifestations of CAD include [4,5]:

- **The asymptomatic period**: The process of atherosclerosis does not cause symptoms. Furthermore, patients who do not have severe coronary artery stenosis may have no symptoms, despite the presence of atherosclerotic lesions in the coronary arteries [6].
- **Stable angina**: The appearance of angina pain either during physical activity or during intense emotional stress. Stable angina is generally a relatively benign clinical condition and usually offers the opportunity to select and apply the appropriate treatment [7].
- **Unstable angina**: The appearance of angina pain at rest. This is a more dangerous form of coronary artery disease, which is why it has been described as pre-infarction angina. It is clear that such an unstable condition must be treated with hospitalization so that the administration of appropriate treatment can be commenced in order to avoid undesirable progression to myocardial infarction [8].
- **Acute myocardial infarction**: This is the necrosis of an area of the heart muscle that manifests itself with typical angina, which, however, is prolonged, does not stop with rest and lasts more than half an hour. The immediate transfer of the patient to a hospital is imperative, because only in a specialized area and by specialized personnel can such a serious medical problem be treated with the greatest possible rate of success [9].
- **Sudden cardiac death**: This is the most dramatic manifestation of the entire clinical spectrum of coronary artery disease [10].

Coronary artery disease is mainly due to atherosclerosis of the coronary arteries. The cause of atherosclerosis is not singular; for it to occur, many factors work together, i.e., it is a multifactorial disease. The factors that all act together as the cause of atherosclerosis are called risk factors or predisposing factors and are the following: gender, age, heredity, hypercholesterolemia, smoking, hypertension, obesity and sedentary lifestyle, diabetes mellitus, metabolic syndrome, chronic renal failure and stress [11,12].

The prevention of heart disease and, therefore CAD lies mainly in changing lifestyles and adopting healthier habits. A balanced diet, exercise and getting rid of bad habits will keep the arteries strong and clean of atherosclerotic plaques. More specifically, some ways to help improve the health of the cardiovascular system are the following: smoking cessation, regular physical exercise, control of blood pressure, low cholesterol and lack of diabetes, maintaining a stable body weight, reducing stress, eating a Mediterranean diet rich in fruits and vegetables, avoiding salt, consuming of foods rich in fibre and limiting alcohol consumption [13,14].

Nowadays, medicine has a variety of modern diagnostic tests, which, in cooperation with Information technology and, especially, the fields of artificial intelligence (AI) and machine learning (ML), in the hands of cardiologists are powerful weapons for the prevention or diagnosis of coronary artery disease. ML techniques now play an important role in the early prediction of disease complications in diabetes (as classification [15,16] or regression tasks for continuous glucose prediction [17,18]), cholesterol [19,20], hypertension [21,22], chronic obstructive pulmonary disease (COPD) [23], COVID-19 [24], stroke [25], chronic kidney disease (CKD) [26], liver disease (LD) [27], sleep disorders [28,29], hepatitis C [30], cardiovascular diseases (CVDs) [31], lung cancer [32], and metabolic syndrome [33] etc. In particular, the long-term risk prediction of CAD will concern us in the context of this study. The main contributions of the present research work are the following:

- Data preprocessing is achieved with the SMOTE. In this way, the instances of the dataset are distributed in a balanced way, allowing us to design robust classification models to ensure a highly accurate prediction of CAD occurrence.
- Features’ importance evaluation is performed considering two commonly used methods, the gain ratio and random forest methods. This analysis is made using the initial unbalanced data and those obtained after class balancing using SMOTE.
- Experimental evaluation is performed with various ML models, after the use or not of SMOTE, evaluating and comparing them in terms of accuracy, precision, recall and AUC. The experimental results indicated that the stacking ensemble model after SMOTE, with 10-fold cross-validation, prevailed over the other ones, constituting the main proposition of this research paper.

The rest of the paper is organized as follows. In Section 2, a dataset description and analysis of the methodology followed are made. Additionally, in Section 3, we discuss the acquired research results. Then, Section 4 discusses the relevant works with the subject under consideration. Finally, conclusions and future directions are outlined in Section 5.

## 2. Materials and Methods

In this section, an overview of the dataset we relied on is carried out, the methodology followed is captured, details of the experimental setup are noted, and brief descriptions of the ML models we experimented with and their evaluation metrics are outlined.

### 2.1. Dataset Description

In this research paper, we used a publicly available dataset [34]. The present dataset includes 3655 instances. It has 15 features, 7 of which are nominal and 8 numerical. Specifically nominal are gender [35], education [36], current smoker [37], blood pressure medication (BPMeds) [38], prevalent stroke (prevStroke) [39], prevalent hypertension (prevHyp) [40] and diabetes [41], while numerical are age [9], cigarettes per day (cigs per day) [42], total cholesterol (totChol) [43], systolic blood pressure (sysBP) [44], diastolic blood pressure (diaBP) [45], body mass index (BMI) [46], heart rate [47] and glucose [48]. The target class, denoted as CAD, is binary and refers to coronary artery disease occurrence or not.

Further statistical details about the features in terms of the target class labels are presented in Table 1. More specifically, the number of participants who have been diagnosed with CAD is 556 (15.2%). Furthermore, the number of women is 2033 (55.6%), while the number of men is 1622 (44.4%). The age of the participants varies from 32 to 70 years.

### 2.2. Methodology

The following subsections emphasize the methodology followed in order to evaluate the ML models we experimented with.

#### 2.2.1. CAD Risk Prediction

The long-term risk prediction of coronary artery disease is formulated as a classification problem with two possible classes c = “CAD” or c = “non-CAD”. The trained ML models will be able to predict the class of a new unclassified instance either as CAD or non-CAD, based on the input features’ values, and thus predict the risk of coronary artery disease.

#### 2.2.2. Data Preprocessing

The accurate identification of CAD and non-CAD instances may be impacted by the unbalanced distribution of the instances in the two classes. Here, an oversampling method is applied, namely SMOTE [49], which is based on the K-Nearest Neighbors (KNN) [50] classifier with K=5 and creates synthetic data [51] on the minority class (see Algorithm 1). The instances in the CAD class are oversampled, such that the subjects in the two classes are uniformly distributed. After the application of SMOTE, the dataset becomes balanced, the number of participants is 6198 and the class variable includes 3099 CAD and 3099 non-CAD instances.
**Algorithm 1:** SMOTE**Input**: *M* (number of samples in the minority class), *N* (% ratio of synthetic minority samples for class balancing), *K* (number of nearest neighbors), $s_{syn}$ synthetic instance;Choose randomly a subset S of the minority class data of size $S=\frac{N}{100}M$ (synthetic samples in the minority class) such that the class labels are uniformly distributed; **for all**$s_{i}\in S$**do**    (1)Find the *K* nearest neighbors;    (2)Randomly select one of KNN, called ${\hat{s}}_{i}$;    (3)Calculate the distance $d_{i,k}={\hat{s}}_{i}-s_{i}$ between the randomly selected NN ${\hat{s}}_{i}$ and the instance $s_{i}$;    (4)The new synthetic instance is generated as $s_{syn}=s_{i}+\delta d_{i,k}$ (where δ=rand(0,1) is a random number between 0 and 1);**end for**Repeat steps number 2–4 until the desired proportion of minority class is met.

The number of women is 2805 (45.3%), while the number of men is 3393 (54.7%). Finally, statistical details about the features in the balanced data are outlined in Table 2.

#### 2.2.3. Features Analysis

In the context of this subsection, our aim is to investigate the importance of the features that represent the instances of the dataset. Two different methods were used: gain ratio and random forest.

First, we employed the gain ratio (GR) method [52] to measure the importance of the features in predicting the target class, calculating it as $GR\left(X_{i}\right)=\frac{H\left(C\right)-H\left(C|X_{i}\right)}{H(X_{i})}$, for i=1,2,⋯,15. In the previous equation, the denominator is the entropy of feature $X_{i}$ defined as $H\left(X_{i}\right)=-\sum_{x_{i}\in V_{i}}p\left(x_{i}\right)log_{2}\left(p\left(x_{i}\right)\right)$ (with $V_{i}$ be the set of different values and $p_{x_{i}}$ denotes the probability of state $x_{i}$ of feature $X_{i}$). Furthermore, the left term in the nominator is the entropy of class variable *C* defined as $H\left(C\right)=-\sum_{c\in C}p\left(c\right)log_{2}\left(p\left(c\right)\right)$ (with p(c) being the probability of state c∈C={CAD,Non−CAD}). Finally, the right term in the nominator is the conditional entropy of feature $X_{i}$ given the *C* which is calculated as $H\left(C|X_{i}\right)=-\sum_{c\in C}\sum_{x_{i}\in V_{i}}p\left(c|x_{i}\right)log_{2}\left(p\left(c|x_{i}\right)\right)$ (where $p(c|x_{i})$ is the related conditional probability of state *c* given value $x_{i}$).

**Figure 1:**
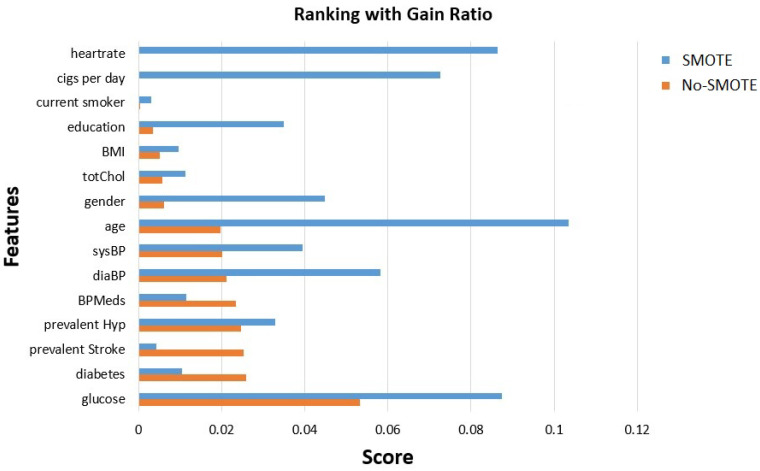
Gain ratio features’ importance evaluation before and after SMOTE.

In Figure 1, we exploited the GR method to capture the features’ order of importance before and after the use of SMOTE. We observed that after SMOTE, heart rate and cigarettes per day were categorized third and fourth in order, respectively, which without SMOTE were last in order with zero scores.

Random forest is a popular machine-learning algorithm characterized by high-accuracy predictive ability, low overfitting (better generalization), and easy interpretability. Feature selection using random forest is categorized as an embedded method that achieves a ranking of importance by the Gini impurity index. Gini impurity is computed at every node split during the construction of a decision tree and measures the quality of the split in terms of separating the samples of the different classes in the specific node. The higher the increment in leaf purity, the higher the importance of the feature. This is applied for each tree and averaged among all the trees normalized to 1. So, the sum of the importance scores calculated by a random Forest is 1. Gini impurity index is computed based on Equation [53]:

(1) $$G=\sum_{i=1}^{c}p_{i}{\left(1-p_{i}\right)}^{2},$$

where *c* denotes the number of classes and $p_{i}$ is the probability of a sample being categorized in class *i*.

**Figure 2:**
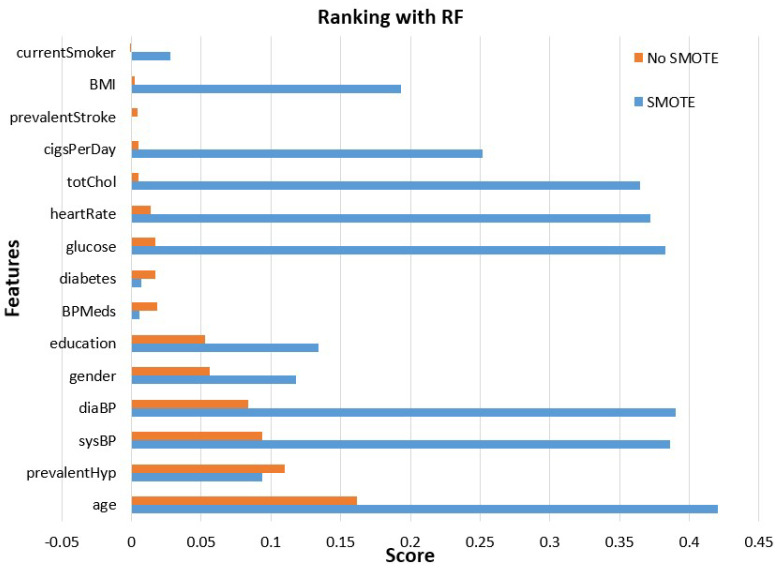
RF features’ importance evaluation before and after SMOTE.

In Figure 2, features’ importance is computed based on random forest, which exploits (1). Observing this figure, the features’ importance was essentially increased, and some of them, such as BMI, cigarettes per day, and heart rate were elevated from the bottom to the top of the hierarchy. Both in the case of random forest, most of the features’ importance was enhanced except for diabetes, stroke prevalence and blood pressure medication (BPMeds). For the models’ training and testing, all of these features were exploited.

### 2.3. Machine Learning Models

In this research article, we experimented with various ML models to discover which one outperforms the others by evaluating their prediction performance. Specifically, we focused on naive Bayes (NB) [54], which assigns an instance to that class for which the conditional probability of the features’ set given class label is maximized, and logistic regression (LR) [55], which are probabilistic classifiers. Furthermore, we used a decision-tree-based model, especially J48 [56]. From ensemble ML algorithms, bagging [57], random forest (RF) [58], rotation forest (RotF) [59], voting [60] and stacking [61] were exploited. Furthermore, a fully connected class of feedforward artificial neural network (ANN), i.e., multilayer perceptron (MLP) [62], and KNN, a distance-based classifier, were evaluated. Finally, in Table 3, we illustrate the optimal parameters’ settings of the ML models that we experimented with.

### 2.4. Evaluation Metrics

In order to evaluate the ML models’ performance, we relied on the accuracy, precision, recall and AUC metrics [63]. The confusion matrix consists of the elements true positive (TP), true negative (TN), false positive (FP) and false-negative (FN). The aforementioned metrics are defined as follows:

- Accuracy: Summarizes the performance of the classification task and measures the number of correctly predicted instances out of all the data instances.
  (2) $$\mathrm{Accuracy}=\frac{\mathrm{TN}+\mathrm{TP}}{\mathrm{TN}++\mathrm{FN}+\mathrm{TP}+\mathrm{FP}}$$
- Precision: Shows the ratio of positive subjects in relation to true and false positive subjects.
  (3) $$\mathrm{Precision}=\frac{\mathrm{TP}}{\mathrm{TP}+\mathrm{FP}}$$
- Recall: Corresponds to the proportion of participants who were diagnosed with CAD and were correctly considered positive, concerning all positive participants.
  (4) $$\mathrm{Recall}=\frac{\mathrm{TP}}{\mathrm{TP}+\mathrm{FN}}$$
- In order to evaluate the distinguishability of a model, the AUC is exploited. It is a metric that varies in [0, 1]. The closer to one, the better the ML model performance is in distinguishing CAD from non-CAD instances.

### 2.5. Experimental Setup

For the evaluation of our ML models, we relied on the Waikato environment for knowledge analysis (Weka) [64]. In addition, the experiments were performed on a computer system with the following specifications: 11th generation Intel(R) Core(TM) i7-1165 G7 @ 2.80GHz, RAM 16 GB, Windows 11 Home, 64-bit OS and x64 processor. We applied 10-fold cross-validation in order to measure the ML models’ efficiency in the balanced dataset of 6198 instances after SMOTE, and in the unbalanced dataset of 3655 instances without SMOTE.

## 3. Results

The purpose of our evaluation is to highlight the role of the SMOTE technique in terms of developing ML models of high reliability and accuracy. In this direction, we experimented with well-known ML models, such as NB, LR, RotF, MLP, KNN, J48, bagging, RF, voting and stacking, evaluating them in terms of accuracy, recall, precision and AUC after 10-fold cross-validation with and without the use of SMOTE.

Specifically, the initial dataset includes 3655 instances. The number of participants who have been diagnosed with CAD is 556 (15.2%), while the non-CAD participants are 3099 (84.8%). According to Table 4 and without the application of SMOTE, the ML models we experimented with have quite high accuracy rates (as this metric captures the overall classification performance in both states of the class label) and less good rates in terms of AUC. AUC is a measure that shows the separation ability of a model among the distributions of CAD and non-CAD instances. The smaller their overlap is, the higher the AUC values will be. In the current dataset, the AUC values without SMOTE reveal that the models have a chance between 55.4% (KNN) and 71.3% (RotF) of being able to distinguish between CAD and Non-CAD classes. Moreover, focusing on the values of the Recall metric, which captures how many of the samples belonging to the CAD class were correctly classified, these ones are significantly low, ranging from 4.5% (bagging) to 31.8% (NB).

Furthermore, from Table 4, we see that after applying SMOTE, the ML models achieved very high-performance metrics. Focusing on the acquired experimental outcomes of the recall metric, its superiority over the No-SMOTE case is significant due to the reduction in false-negative predictions. This is of great importance and plays a decisive role in the design of efficient ML models and techniques. The ratio of correctly recognized CAD samples ranges from 74.2% (LR) to 87.6% (stacking). The accuracy was less enhanced by the application of SMOTE, while the highest improvement of 10.6% is observed by the NB classifier.

**Figure 3:**
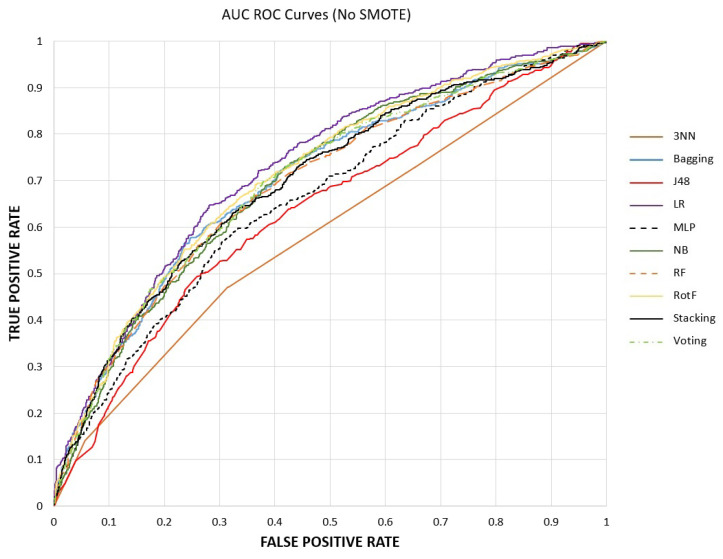
Performance Evaluation with AUC ROC Curves before SMOTE.

**Figure 4:**
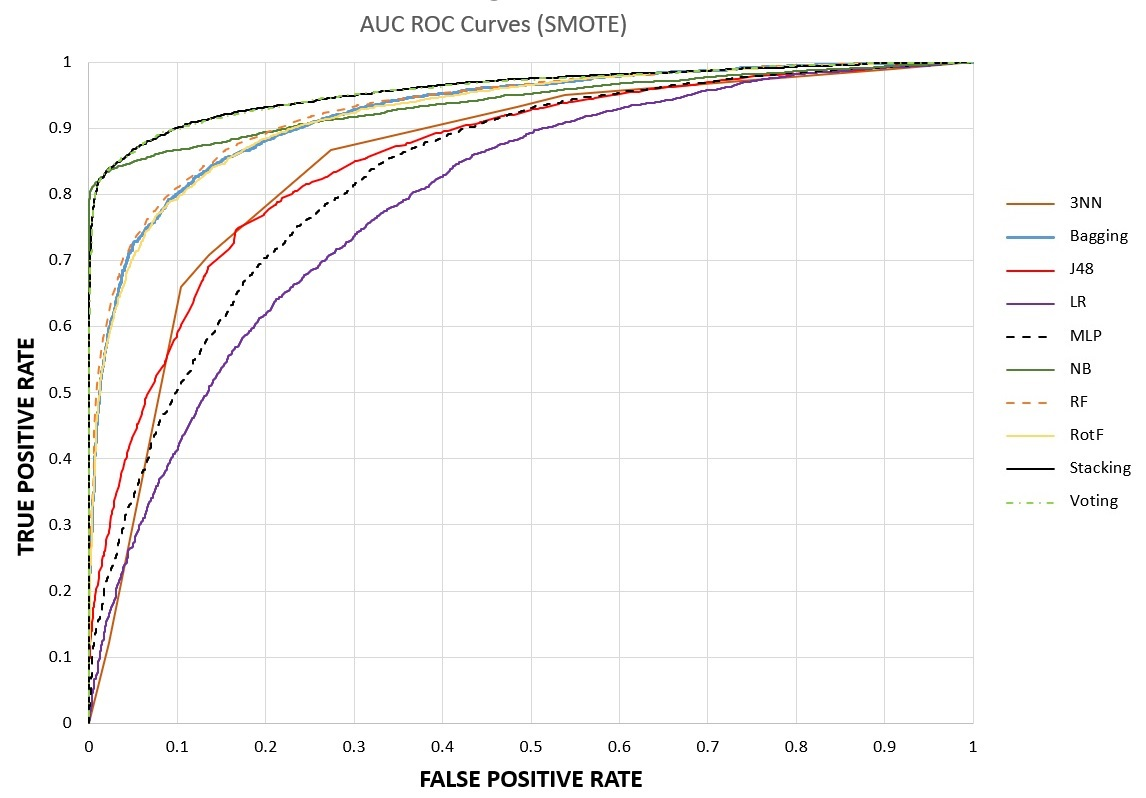
Performance Evaluation with AUC ROC Curves after SMOTE.

To further interpret the classification performance of ML models, AUC–ROC curves are plotted in Figure 3 and Figure 4, before and after the application of SMOTE. These are probability curves that capture the relationship between the true positive rate (TPR or recall) and the false positive rate (FPR), where FPR is defined as the ratio $\frac{FP}{FP+TN}$. As the results indicate, the SMOTE benefited most of the models by significantly improving the recall of the CAD class; thus, the AUC curves of ensemble models became more abrupt, starting from lower values of FPR and attaining one. As a final note, it is observed that after class balancing, stacking and voting have identical AUC curves, with a small lead in stacking in all metrics.

Concluding the results section, we should highlight that the stacking ensemble model after SMOTE with 10-fold cross-validation prevailed over the other models, achieving an accuracy of 90.9%, a precision of 96.7%, a recall of 87.6% and an AUC equal to 96.1%.

## 4. Discussion

In this section, a brief description of relevant works on coronary artery disease risk prediction is provided with the contribution of ML models and techniques.

First, the authors in [65] tested ten traditional ML algorithms. They also introduced a new optimization technique called the N2 Genetic optimizer. The experiments demonstrated that N2 Genetic-nuSVM provided an accuracy of 93.08% and an F1 score of 91.51% when predicting CAD outcomes among the patients included in the Z-Alizadeh Sani dataset.

Similarly, the authors in [66] used the publicly available Z-Alizadeh Sani dataset, which contains random samples of 216 cases with CAD and 87 normal controls with 56 different features. Five different supervised classification ML algorithms, LR, a classification tree with bagging (bagging CART), RF, SVM, and KNN, were applied. Finally, the results indicate that the SVM model is able to predict the presence of CAD more effectively and accurately than other models, with an accuracy of 89.4%, a sensitivity of 94.3%, a specificity of 78.2%, and an AUC of 88.7%.

In addition, the authors in [67] compare the accurate prediction results of NB and SVM in order to predict CAD in a timely manner. This research paper uses two types of datasets (noisy and less noisy images along with numerical features), where the models experimented with them. The NB model has lower accuracy compared to the SVM in both cases.

In [68], the authors applied ML algorithms, including SVM, KNN, RT, RF, NB, gradient boosting (GB) and LR, on a dataset obtained in the two General Hospitals in Kano State, Nigeria for the prediction of CAD. In terms of accuracy, the random forest model emerged as the best model with 92.04%; for specificity, the NB model was the best, with 92.40%. For sensitivity, the SVM model was the best, with 87.34%, and for the AUC, the best model was the RF model, with 92.20%.

The research study in [69] aimed to improve the accuracy of CAD diagnosis by selecting the most significant features. For this purpose, several ML models such as the RT, the C5.0 DT and the SVM, were evaluated. The RT showed promising results, achieving the highest accuracy of 91.47%.

Moreover, in [70], the LR, SVM and ANN algorithms are the points of interest. In order to evaluate the results, the accuracy and AUC scores have been performed using the 10-fold cross-validation. The SMOTE technique has been used to balance the dataset. The ANN achieved the highest accuracy of 93.35% and an AUC of 98% for CAD prediction.

Furthermore, the same methodology is followed by the authors in [71]. Three feature selection methods have been used on 13 input features from the Cleveland dataset with 297 entries, and 7 were selected. Specifically, SVM, NB and KNN using 10-fold cross-validation were applied for CAD prediction. The NB classifier performs the best on this dataset, achieving an accuracy of 84%.

Furthermore, the authors in [72,73,74] experimented with the same dataset [34] as the current study. In [72], the neural network is the algorithm that yielded the greatest AUC in R-studio when excluding observations in which there was at least one missing value (AUC = 71%). When the data was analyzed in RapidMiner, the best algorithm was SVM (AUC = 75%).

The study in [73] applied LR, NB, DT, KNN, SVM and RF in order to predict whether a subject runs a risk of future development of CAD or not in the next ten years. The RF model outperformed the other models with an accuracy of 91.1%, a precision of 64.3% and a recall equal to 6.4%. In [74], work suggested the cloud RF (C-RF) model, which prevailed compared to CART (classification and regression tree), SVM and CNN, with accuracy and an AUC of 85%, similarly.

Here, in the balanced dataset after SMOTE, we exploited more efficient schemes to design the desired classification models, with an emphasis on ensemble techniques. Furthermore, we further validated the expected performance of ensemble models with a graphical illustration of the AUC–ROC curves. To sum up, comparing the performance of [72,73,74], our proposed trained and tested classifier (i.e., stacking) presents an accuracy of 90.9 %, a precision of 96.7%, a recall of 87.6% and an AUC equal to 96.1% after SMOTE with 10-fold cross-validation, thus confirming its high accuracy rates.

## 5. Conclusions

Cardiovascular disease remains the leading cause of death despite significant progress in medical science and contains a wide range of diseases, including all pathological changes involving the heart and/or blood vessels. The long-term risk prediction of CAD disease was the topic under consideration in this research. Furthermore, the features’ importance evaluation, based on the gain ratio and RF, was performed. Through risk factor monitoring and analysis, personalized guidelines and interventions can be suggested to prevent CAD occurrence. Such an analysis can help medical experts regularly reassess underlying risks, and even if CAD occurs, they can provide patients with novel guidelines and treatments based on individual patient characteristics, that may enhance their daily life, increase life expectancy and restrict mortality.

Furthermore, experimental evaluation with various ML models, including NB, LR, RotF, MLP, KNN, J48, bagging, RF, voting and stacking with 10-fold cross-validation, after the use or not of SMOTE, was made. Comparing the ML models in terms of accuracy, recall, precision and AUC, the reliability of the SMOTE technique was demonstrated. The stacking ensemble model after SMOTE with 10-fold cross-validation was the model that prevailed over the other ones, achieving an accuracy of 90.9%, a precision of 96.7%, a recall of 87.6%, and an AUC equal to 96.1%; this constitutes the main proposition of this paper. In future work, we aim to extend the machine learning framework by using deep learning methods and comparing the results based on the aforementioned metrics.

## Figures and Tables

**Table 1:** Numerical and nominal features’ description in the initial dataset before SMOTE.

Attribute	Description			Attribute	Description
	Min	Max	Mean ± stdDev	**Gender**	male (1622), Female (2033)
**Age**	32	70	49.5 ± 8.56	**Education**	PhD (423), BSc (1100), High School (1526), MSc (606)
**Cigs/day**	0	70	9 ± 11.92		
**totChol**	113	464	236.8 ± 43.69	**Current** **smoker**	Yes (1788), No (1867)
**SysBP**	83.5	295	132.3 ± 22.1		
**DiaBP**	48	142.5	82.9 ± 11.97	**BPMeds**	Yes (111), No (3544)
**BMI**	15.54	56.8	25.8 ± 4.07	**prevStroke**	Yes (21), No (3634)
**Heart rate**	44	143	75.7 ± 11.99	**prevHyp**	Yes (1138), No (2517)
**Glucose**	40	394	81.8 ± 23.89	**Diabetes**	Yes (98), No (3557)

**Table 2:** Numerical and nominal features’ description after SMOTE.

Attribute	Description			Attribute	Description
	Min	Max	Mean ± stdDev	**Gender**	Male (3393), Female (2805)
**Age**	32	70	51.5 ± 8.34	**Education**	Phd (665), BSc (1693), High School (3198), MSc (642)
**Cigs/day**	0	70	9.4 ± 11.79		
**totChol**	113	464	240.5 ± 44.18	**Current smoker**	Yes (2803), No (3395)
**sysBP**	83.5	295	136.8 ± 23.8		
**diaBP**	48	142.5	84.7 ± 12.59	**BPMeds**	Yes (111), No (6087)
**BMI**	15.54	56.8	26 ± 3.91	**prevStroke**	Yes (21), No (6177)
**Heart rate**	44	143	75.8 ± 11.45	**prevHyp**	Yes (2335), No (3863)
**Glucose**	40	394	84.3 ± 30.95	**Diabetes**	Yes (183), No (6015)

**Table 3:** Machine learning models’ settings.

Models	Parameters	Models	Parameters
**NB**	useKernelEstimator: False useSupervisedDiscretization: True	**RotF**	classifier: RF numberOfGroups: True projectionFilter: PrincipalComponents
**LR**	ridge = $10^{-8}$ useConjugateGradientDescent: True	**J48**	reducedErrorPruning: False savelnstanceData: True useMDLCorrection: True, subtreeRaising: True binarySplits = True, collapseTree = True
**MLP**	learning rate = 0.1 momentum = 0.2 training time = 200	**Stacking**	classifiers: RF and NB metaClassifier: LR
**KNN**	K=3 Search Algorithm: LinearNNSearch with Euclidean cross-validate = True	**Voting**	classifiers: RF and NB combinationRule: average of probabilities
**RF**	breakTiesRadomly: True numIterations = 500 storeOutOfBagPredictions: True	**Bagging**	classifiers: RF printClassifiers: True storeOutOfBagPredictions: True

**Table 4:** Performance evaluation of ML models in terms of accuracy, precision, recall and AUC metrics.

	Accuracy		Precision (CAD class)		Recall (CAD Class)		AUC	
	No SMOTE	SMOTE	No SMOTE	SMOTE	No SMOTE	SMOTE	No SMOTE	SMOTE
**NB**	0.700	0.906	0.336	0.973	0.318	0.835	0.700	0.941
**LR**	0.754	0.779	0.645	0.710	0.088	0.762	0.729	0.793
**MLP**	0.730	0.798	0.355	0.742	0.146	0.801	0.661	0.833
**3-NN**	0.722	0.796	0.311	0.760	0.140	0.867	0.585	0.854
**RF**	0.748	0.855	0.493	0.844	0.063	0.871	0.693	0.931
**RotF**	0.751	0.845	0.625	0.827	0.054	0.872	0.713	0.925
**J48**	0.714	0.787	0.268	0.777	0.205	0.804	0.636	0.857
**Stacking**	0.747	0.909	0.482	0.967	0.059	0.876	0.698	0.961
**Bagging**	0.748	0.843	0.500	0.827	0.045	0.866	0.702	0.926
**Voting**	0.787	0.908	0.367	0.960	0.187	0.852	0.702	0.958
